# Mid‐Regional Proadrenomedullin Can Be Reliably Measured in Cerebrospinal Fluid to Improve Diagnosis of Central Nervous System Diseases

**DOI:** 10.1002/jcla.70058

**Published:** 2025-05-19

**Authors:** Matteo Furlani, Daniela Visentini, Anna Rosa Cussigh, Fiorenza Pesente, Francesco Janes, Carlo Tascini, Francesco Curcio, Martina Fabris

**Affiliations:** ^1^ Department of Medicine (DMED) University of Udine Udine Italy; ^2^ Institute of Clinical Pathology, Department of Laboratory Medicine, Azienda Sanitaria Universitaria Friuli Centrale (ASU FC) Udine Italy; ^3^ Clinical Neurology, Azienda Sanitaria Universitaria Friuli Centrale (ASU FC) Udine Italy; ^4^ Infectious Diseases Clinic, Azienda Sanitaria Universitaria Friuli Centrale (ASU FC) Udine Italy

**Keywords:** adrenomedullin, central nervous system diseases, cerebrospinal fluid, MR‐proADM, vasoactive peptide

## Abstract

**Background:**

Adrenomedullin (ADM) is a potent hormone‐like peptide rapidly induced by hypoxia and inflammatory cytokines in the early stages of sepsis. For this reason, the dosage of its more stable precursor fragment called mid‐regional (MR)‐proADM is currently recommended to assist in triaging patients in the emergency department. Since MR‐proADM dosage is currently only approved for use in plasma, we validated its dosage in cerebrospinal fluid (CSF) samples to improve the diagnosis of central nervous system (CNS) diseases.

**Methods:**

MR‐proADM concentrations were measured in samples using a fully automated platform (Brahms Kryptor Gold Analyzer, Thermo Scientific, Germany), applying the same analytical conditions in plasma and CSF samples, to finally set up an accurate laboratory protocol to validate its dosage in CSF.

**Results:**

MR‐proADM is highly stable in CSF samples stored at room temperature for up to 48 h, allowing it to be measured with confidence also in CSF samples that may be left on the bench for several hours. In addition, the repeatability and within‐laboratory precision of the MR‐proADM assay using CSF samples appeared equal to or better than those obtained by the manufacturer using plasma samples, allowing the use of this assay, with high precision, also for CSF samples.

**Conclusion:**

The reliable measure of MR‐proADM in CSF and the role of this molecule in CNS will allow its introduction in the diagnostic process of infectious, inflammatory, and degenerative neurological diseases.

## Graphical abstract

Numerous evidences suggest that Adrenomedullin represents an important regulator of endothelial homeostasis also in the central nervous system and may have a role in infectious, inflammatory, and degenerative neurological diseases. Mid‐regional proadrenomedullin (MR‐proADM) can be reliably measured in cerebrospinal fluid using commercially available methods. MR‐proADM is a promising biomarker to improve the diagnostic work‐up of neurological diseases.

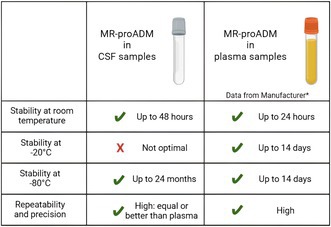

## 1 Introduction

Adrenomedullin (ADM) is a member of the calcitonin gene‐related peptide (CGRP) family of proteins [1] that was isolated for the first time from human pheochromocytoma cells and exerts an important hypotensive effect [2].

**FIGURE 1:**
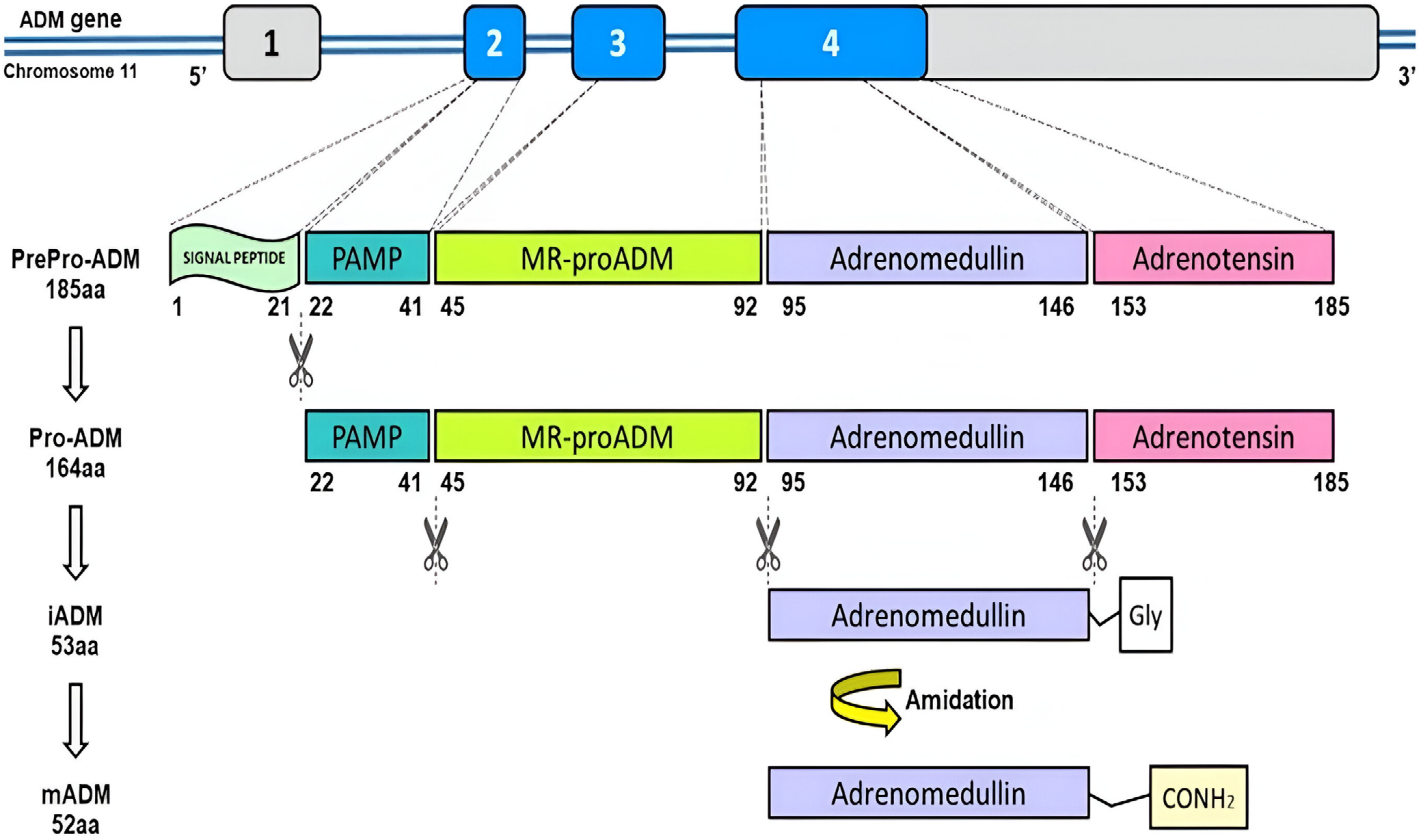
Adrenomedullin (ADM) biosynthesis. The expression of human ADM gene leads to the production of the precursor protein prepro‐ADM. Further post‐translational processing generates pro‐ADM, consisting in the bioactive proadrenomedullin N‐terminal 20 peptide (PAMP), mid‐regional pro‐ADM (MR‐proADM), adrenotensin (ADT) and immature ADM (iADM). Enzymatic amidation converts iADM into the mature form of ADM (mADM) [7].

As well as other biologically active peptides, ADM is evolutionarily conserved and widely distributed and expressed throughout mammalian tissues, including the brain [3]. Its expression was demonstrated in almost all cell types, including neurons and glial cells [4]. The expression of ADM is induced by hypoxia and inflammatory cytokines, such as tumor necrosis factor‐α (TNF‐α) and interleukin 1α (IL‐1α), whereas interferon‐γ (IFN‐γ) and t*ransforming growth factor β 1* (TGF‐β1) downregulate ADM transcription [5]. In addition to cytokines, other factors such as lipopolysaccharides, endotoxins, and physical stress also impact the synthesis and release of ADM [6]. Similar to many other hormones, ADM is synthesized as a large precursor known as prepro‐ADM, consisting of 185 amino acids (Figure 1) [8]. This precursor is subsequently converted into pro‐ADM, a 164 amino acid peptide, by cleaving the N‐terminal signal peptide. Pro‐ADM is then split into three vasoactive peptides [proadrenomedullin N‐terminal 20 peptide (PAMP), adrenotensin pro‐ADM153‐185 (ADT) and immature ADM (iADM)], and one inactive fragment, the Mid‐regional proADM (MR‐proADM) [9], for which no physiological function has been identified. The reliability of ADM measurement in body fluids is limited by its very low picomolar concentrations due to its rapid degradation by proteases and binding to AMBP‐1, also known as complement factor H, so its half‐life is very short (only 22 min) [10, 11]. In contrast, the biologically inactive MR‐proADM is more stable and has a longer half‐life. Since ADM and MR‐proADM are produced 1:1 during post‐transcriptional processes, measuring MR‐proADM is a reliable estimation of ADM concentration [12].

Recent studies have shown that ADM, as a marker of endothelial dysfunction, is rapidly induced in the initial stages of sepsis [13] and becomes extremely elevated when sepsis progresses to multiple organ failure [14]: in such a way, it represents a convenient biomarker for early identification of patients with severe infections progressing to multiple organ failure [15, 16, 17, 18, 19]. On the other hand, it can be used to assist triaging in the emergency department, allowing safely discharged patients with suspected infections [20, 21].

The exceptionality of MR‐proADM also derives from its kinetic profile, since it is more rapidly produced compared to C‐reactive protein (CRP) and procalcitonin (PCT), consistent with previous studies that indicate MR‐proADM as a more accurate biomarker than CRP and PCT in stratifying disease severity and predicting treatment response [22, 23].

All these exceptional features can be translated to the central nervous system (CNS).

In fact, as concerns the neurological tissues, ADM directly promotes cellular growth and neo‐angiogenesis and acts as a neuromodulator, disclosing an important protective effect against brain injuries [24]. ADM is highly expressed in the spinal cord and a recent study showed that dorsal root ganglia and spinal motor neurons both express and are targets of ADM action, providing the possible mechanisms to explain the beneficial role of ADM in protecting, survival, and regeneration of sensory and motor neurons [25]. The same autocrine loop active in neurons is also present in astrocytes and cerebral endothelial cells, since they released ADM and express its functional receptor on the cell surface [26]. The first studies about the role of ADM in the context of brain injury gave conflicting results, describing both beneficial and detrimental effects. Only successive genetic experiments provide an explanation. In a permanent focal ischemia mice model, when ADM is knocked out in neurons, the infarct volume and the consequent brain damage were increased, demonstrating that in neurons ADM exerts a neuroprotective action mediated by inducible NO synthase, matrix metalloprotease 9, and COX‐2 [27]. On the other hand, when ADM is knocked out in endothelial cells, infarct volume and brain damage were reduced, demonstrating that ADM in the endothelium has a harmful effect on brain injury [28]. In fact, patients with stroke disclosed ADM overexpression and higher ADM expression correlates with stroke severity and poor prognosis [28]. Thus, ADM exerts different biological effects in different cell contexts and, independently or collectively, contributes to brain vascular pathology.

For example, in neurons, increased levels of ADM and its related peptides may induce the destabilization of cytoskeletal proteins, resulting in axon transport impairment and synaptic failure that finally cause neuronal degeneration as in Alzheimer's disease (AD) [29]. In fact, AD patients present increased brain ADM, and some authors proposed to measure ADM or its precursor fragment, MR‐proADM, in blood as a marker to predict progression from pre‐dementia to clinical AD [30]. In addition, reducing ADM expression may represent a new therapeutic option to prevent and treat AD.

On the other hand, ADM produced by cerebral vascular endothelium plays a key role in restoring endothelial stability and permeability following severe infection, since ADM is involved in the physiological maintenance of the blood–brain barrier [31].

Currently available assays for MR‐proADM are only certified for plasma samples [32]. To date, there are no reports about MR‐proADM routine testing in cerebrospinal fluid (CSF). Based on available data about its role in CNS, MR‐proADM may represent a prognostic biomarker of CNS infectious, inflammatory, and degenerative diseases. To be widely and appropriately measured in a diagnostic setting, MR‐proADM testing in CSF needs to be validated according to international guidelines [33]. For this reason, we designed an accurate study to validate the analysis of MR‐proADM in CSF using the method currently available in our laboratory.

## 2 Methods

### 2.1 MR‐proADM Assay on Kryptor Analyser (THERMO SCIENTIFIC, Hennigsdorf, Germany)

MR‐proADM concentrations were measured in CSF using the B.R.A.H.M.S. MR‐proADM fluoroimmunoassay on the Brahms Kryptor Gold Analyzer (THERMO SCIENTIFIC, Hennigsdorf, Germany), which is a fully automated homogeneous phase random access platform. The system uses the TRACE (Time‐Resolved Amplified Cryptate Emission) technology to measure the fluorescence signal emitted by an immunocomplex. MR‐proADM results are expressed in nmol/L. The declared linearity in plasma EDTA samples ranges from 0.21 to 100 nmol/L, with a maximum bias of ±20%. The manufacturer's reference range for EDTA plasma samples is < 0.56 nmol/L, corresponding to the 97.5th percentile of a control series, which disclosed a median value of 0.38 nmol/L. The Limit of Detection (LoD) is 0.09 nmol/L, while the Limit of Quantitation (LoQ) is 0.21 nmol/L (IFU_B.R.A.H.M.S. MR‐proADM KRYPTOR assay; 07 April 2022; Thermo Scientific, Hennigsdorf, Germany). This MR‐proADM immunoassay is currently certified only for plasma‐EDTA blood samples, and the manufacturer recommends immediate centrifugation to preserve molecular stability at room temperature (18°C–25°C) or at 2°C–8°C up to 24 h. Afterwards, samples should be aliquoted and frozen at −20°C or below. Plasma samples can be kept frozen up to 14 days, and the analyte is considered stable for up to 4 freeze/thaw cycles.

### 2.2 CSF Sample Collection and Study Protocol

The main focus of our study was to assess the stability of MR‐proADM in CSF and the reliability of its measure using the B.R.A.H.M.S. MR‐proADM method. In the absence of CSF normal samples, to validate the above‐mentioned method, we recovered the residual CSF samples from the diagnostic analyses in our Laboratory. The study was conducted in accordance with the Declaration of Helsinki (as revised in 2013). Only anonymized leftovers samples from routine clinical practice were used and their use was not subjected to ethics review, according to the International Standard ISO 15189 and to the Italian legislation (Authorization of the Privacy Guarantor No. 9, 12th of December 2013), following the Food and Drug Administration OBM Control No. 0910‐0582 “Guidance of informed consent for in vitro diagnostic device studies using leftover human specimen that are not individually identified.”

According to the recommendations in plasma, MR‐proADM was measured in clear CSF samples after centrifugation to avoid interference by cells or other microparticles. Bloody CSF samples were discarded. The first step was aimed at assessing MR‐proADM stability in CSF as a function of different storage temperatures and storage time after collection. To test room temperature stability in CSF, we measured MR‐proADM in 10 fresh samples (T0) and after 12, 24, and 48 h (T1, T2, T3) on the bench (25°C). To test recovery after storage at −20°C, we measured MR‐proADM concentration in 29 CSF samples stored frozen for less than 15, 16 to 60, and more than 60 days. Finally, to test recovery after storage at −80°C, we measured MR‐proADM concentration in 25 CSF samples before and after a variable period of storage at −80°C. The second step of our study was to evaluate the analytical performance of the B.R.A.H.M.S. MR‐proADM method using CSF samples. For this purpose, we evaluated the repeatability and the within‐laboratory precision of this assay. Due to the small amount of CSF volume obtained from each residual sample, 58 CSF samples were pooled in 3 subgroups according to their MR‐proADM values, thus creating 3 pools of about 5 mL volume each, characterized by low, medium, and high MR‐proADM concentration. These pools were finally stored at −20°C.

To evaluate repeatability and within‐laboratory precision, we performed a protocol based on the guidelines of Clinical and Laboratory Standards Institute (CLSI) document EP05‐A3 [33] and on the Italian guidelines for immunoassay determinations in biological fluids [34]. The CLSI EP05‐A3 document suggests two different protocols to evaluate precision performances. The standard protocol is the “20 × 2 × 2 model,” in which samples must be measured up to 20 days, with 2 runs per day and 2 replicates. A second model, the “5 × 5 × 3,” is also proposed by the EP05‐A3 document. This model is based on 5 days, with 5 replicates per day, using at least 3 instruments, and permits evaluation of the reproducibility among different instruments, the repeatability, and the within‐laboratory precision [33]. A less extensive protocol for assessing precision performances is suggested in the guidelines for immunoassay determinations in biological fluids, with 5 measurements per day, for 4 consecutive days, using at least 3 different concentrations [34].

Due to the difficulty of obtaining large volumes of CSF and to the availability of only one instrument in the laboratory, we performed a protocol in which 3 different pools, with different concentrations, were measured in 5 replicates, on 5 runs, on a single instrument for 5 days.

In this way, we estimated repeatability and within‐laboratory precision with 25 measurements for each of the three different concentrations of MR‐proADM. Repeatability and within‐laboratory precision are both expressed as coefficients of variation (CV%).

### 2.3 Statistical Analysis

To assess the difference between measures of MR‐proADM in fresh samples and after storage at room temperature, at −20°C, and at −80°C, we used the Wilcoxon matched‐paired T test, considering significant *p* < 0.05.

## 3 Results

### 3.1 MR‐proADM Stability in CSF at Room Temperature

As shown in Table 1, MR‐proADM concentration is highly stable in CSF samples stored at room temperature: after 12, 24, and 48 h the concentration did not differ significantly compared to fresh samples.

**TABLE 1:** Stability at RT.

CSF samples	Fresh (nmol/L)	After 12 h (nmol/L)	After 24 h (nmol/l)	After 48 h (nmol/L)
S1	1.15	1.11	1.15	1.14
S2	1.23	1.23	1.25	1.27
S3	0.92	0.91	0.93	0.93
S4	1.07	1.07	1.09	1.12
S5	0.94	0.96	0.93	0.97
S6	0.98	0.96	0.92	0.99
S7	1.65	1.63	1.67	1.64
S8	0.45	0.45	0.51	0.45
S9	2.67	2.67	2.61	2.74
S10	0.79	0.80	0.80	0.78

*Note:* MR‐proADM concentrations in fresh CSF samples did not differ significantly after 12 (*p* = 0.2918), 24 (*p* = 0.6760), and 48 (*p* = 0.1515) hours storage at room temperature (25°C).

### 3.2 MR‐proADM Stability in CSF Samples Stored at −20°C

As shown in Table 2, as concerns storage at −20°C, MRproADM concentration displayed a heterogeneous behavior, since 12 samples show a limited variation (< 10%), but others decreased significantly, in some cases up to 50%. In these cases, considering the physiological cut‐off validated on plasma (0.56 nmol/L) and the fact that a concentration > 0.87 nmol/L in plasma is an alert value [23], the variation observed at the CSF level for some samples (S7, S17) could shift a result from a clearly pathogenic range to an almost normal range. In one case (S22), we observed an increased value, but it remained in the same clinical range.

**TABLE 2:** Stability at −20°C.

CSF samples	Fresh (nmol/L)	1–15 days (nmol/L)	16–60 days (nmol/L)	> 60 days (nmol/L)	Difference (%)
S1	0.47	0.42			10.6
S2	0.71	0.66			7.0
S3	0.77	0.71			7.8
S4	0.77	0.77			0.0
S5	1.07	0.95			11.2
S6	1.07	0.97			9.4
S7	1.70	0.97			42.9
S8	1.77	1.32			25.4
S9	1.84	1.59			13.6
S10	0.62		0.56		9.7
S11	0.67		0.55		17.9
S12	0.73		0.59		19.2
S13	0.88		0.83		5.7
S14	0.93		0.82		11.8
S15	0.96		0.88		8.3
S16	1.08		0.94		13.0
S17	1.63		0.79		51.5
S18	12.97		12.24		5.6
S19	0.53			0.46	13.2
S20	0.57			0.40	29.8
S21	0.57			0.58	1.8
S22	0.58			0.85	46.6
S23	0.73			0.67	8.2
S24	0.74			0.62	16.2
S25	0.80			0.72	10
S26	0.82			0.69	15.9
S27	0.87			0.75	13.8
S28	1.88			1.46	22.3
S29	5.42			3.95	27.1

*Note:* MR‐proADM concentration in CSF samples stored at −20° appeared statistically different from those measured in fresh samples, both in samples stored less than 15 days (*p* = 0.0078) and in those stored 16–60 days (*p* = 0.0091) or more than 60 days (*p* = 0.0453).

We subdivided the CSF samples in 3 series, based on the storage time frame: 1 to 15 days, 16 to 60 days, and more than 60 days. In samples stored at −20°C for less than 15 days, the mean difference pre and post freezing was 14.2%, while in samples stored from 16 to 60 days it increases to 15.9%, and in samples stored for more than 60 days it increases to 18.6%, so it seems that the level of recovery after freezing depends mainly on the storage time. Anyway, although the concentration measured after storage at −20°C is not accurate, in most cases it remains in the same range (around 0.56 nmol/L, around 1 nmol/L or > 1 nmol/L), preserving the clinical significance.

### 3.3 MR‐proADM Stability in CSF Samples Stored at −80°C

As shown in Table 3, the measurements of MR‐proADM in 25 CSF samples stored for a variable time (less than 6 months, 6–12 months and more than 12 months) at −80°C appeared to be quite stable: half of the samples showed a decrement equal to or less than 10%, and the other half remained between 10% and 20%.

**TABLE 3:** Stability at −80°C.

CSF samples	Fresh (nmol/L)	0–6 months (nmol/L)	7–12 months (nmol/L)	13–18 months (nmol/L)	> 18 months
S1	0.62	0.49			
S2	0.64	0.57			
S3	0.71	0.64			
S4	0.73	0.73			
S5	0.77	0.69			
S6	0.79	0.69			
S7	0.54		0.52		
S8	0.55		0.43		
S9	0.56		0.47		
S10	0.77		0.65		
S11	0.51			0.47	
S12	0.53			0.46	
S13	0.59			0.50	
S14	0.66			0.66	
S15	0.70			0.55	
S16	0.89			0.78	
S17	1.00			0.88	
S18	0.43				0.38
S19	0.51				0.43
S20	0.53				0.52
S21	0.57				0.54
S22	0.59				0.56
S23	0.64				0.64
S24	0.69				0.64
S25	0.75				0.59

*Note:* MRproADM concentration in CSF samples store at −80°C showed a statistically significant decrement as compared to fresh samples (*p* < 0.001). However, the decrement remains around 10% in the majority of cases.

### 3.4 Repeatability and Precision

Repeatability and within laboratory precision were calculated using 25 measurements for every selected range of MR‐proADM concentration (low, medium, high). As shown by CV% reported in Tables 4 and 5, repeatability and within‐laboratory precision at each concentration were equal to or better than those obtained by the manufacturer using plasma samples.

**TABLE 4:** Analytical performance: repeatability.

CSF samples	Mean MR‐proADM (nmol/L)	Repeatability (CV%)	Plasma samples*	Mean MR‐proADM (nmol/L)	Repeatability (CV%)
Pool 1	0.44	3.8	Sample 1	0.23	7.6
Pool 2	0.68	2.5	Sample 2	0.85	2.0
Pool 3	2.96	0.6	Sample 3	2.20	1.2

*Note:* The repeatability, expressed as coefficient of variation (CV%), obtained for MR‐proADM measurements in CSF pooled samples showed results comparable to those reported by the manufacturer in plasma samples*, with comparable mean concentrations (pool 1: low concentration; pool 2: moderate concentration; pool 3: high concentration). The CV% for repeatability was calculated as the ratio between the within‐run standard deviation and the overall media × 100. Within‐run standard deviation was calculated as the average standard deviation of daily analytical sessions.

**TABLE 5:** Analytical performance: precision.

CSF samples	Mean MR‐proADM (nmol/L)	Within‐laboratory precision (CV%)	Plasma samples*	Mean MR‐proADM (nmol/L)	Within‐laboratory precision (CV%)
Pool 1	0.44	4.5	Sample 1	0.23	15.2
Pool 2	0.68	2.9	Sample 2	0.85	10.4
Pool 3	2.96	2.4	Sample 3	2.20	4.9

*Note:* The within‐laboratory precision, expressed as coefficient of variation (CV%), obtained for MR‐proADM measurements in CSF pooled samples, showed better performances than those reported by the manufacturer in plasma samples*, at comparable mean concentrations (pool 1: Low concentration; pool 2: Moderate concentration; pool 3: High concentration). Coefficient of variation for within‐laboratory precision was calculated as the ratio between total standard deviation and the overall media × 100. Total standard variation was calculated as the sum of between‐run standard deviation and within‐run standard deviation multiplied for the (N‐1)/N coefficient, where N is the number of replicates (in our case the coefficient is 4/5).

## 4 Discussion and Conclusions

The review of the literature [24, 25, 26, 27, 28, 29, 30, 31] on the role and prognostic perspectives of ADM also at the level of the central nervous system makes it a promising CSF biomarker, both in the critical patient to suspect a pre‐septic state and in the chronic patient with neuro‐degenerative and/or inflammatory diseases. In order to guarantee a reliable CSF dosage and therefore transferable to the diagnostic setting, it is essential to study the analytical performances of the methods currently available in the market, which are validated only on plasma. In this study, according to analytical procedures indicated by international guidelines, we showed that MR‐proADM can be measured in CSF samples using the same method as for plasma samples, with optimal repeatability and within‐laboratory precision, both in low and in high concentrated samples.

Of note, MR‐proADM remains highly stable in CSF samples stored at room temperature for as long as 48 h, allowing for measurement with confident results also in residual CSF samples, which may be left on the bench for several hours. This is a very good property for a biomarker when it must be introduced into a diagnostic setting. In contrast, the stability of MR‐proADM in CSF samples stored at −20°C is not optimal and appears to be time‐related since the mean recovery decreased from samples stored < 15 days to those stored > 60 days. In the majority of samples, the percentage reduction ranges between 10% and 20%, but in some samples, it was about 50%. However, in general, MR‐proADM concentration remained in the same range, preserving the potential clinical significance. The heterogeneity of the stability obtained in samples stored at −20°C can be explained by the fact that this storage is less controlled and stable than that at −80°C. In fact, samples stored at −80°C showed a very good stability for a long time (at least 24 months in our experience), with a loss of recovery generally less than 10%. Our results suggest that CSF samples stored at −80°C can be used to test MR‐proADM in retrospective studies, while samples stored at −20°C must be used with caution for possible underestimation compared to fresh samples.

In our experience (data not shown) it is advisable to test MR‐proADM in CSF and in plasma at the same time, and to compare these concentrations, to better identify where the major source of altered expression is, in other words, to understand whether the up‐regulation of MR‐proADM is selectively present in the CSF and not the consequence of altered blood–brain barrier permeability.

To facilitate this interpretation, an automatically calculated ratio between CSF and plasma MR‐proADM concentration can be inserted in the medical report. This is particularly useful since the physiological range of CSF MR‐proADM concentration is actually unknown and extremely difficult to estimate, and since healthy CSF samples are rarely available, a lumbar puncture is always performed when there is some kind of CNS disorder. Moreover, it is very likely that ADM concentration in CSF depends on age and sex, as it is closely correlated to brain endothelial dysfunction [26].

In order to overcome these limitations, the measure at the same time CSF and plasma MR‐proADM will facilitate the interpretation of altered CSF values, since plasma concentration may be considered as a patient‐specific normalizer to understand how much the CNS is compromised compared to the systemic bloodstream.

In conclusion, our study showed that MR‐proADM is stable in CSF samples and it can be measured by automated methods with the same analytical performance validated in plasma. This will offer the possibility of carrying out large‐scale studies to set up cut‐off values and confirm the promising opportunities of this biomarker in central nervous system acute and chronic diseases.

## Conflicts of Interest

The authors declare no conflicts of interest.

## Data Availability

The data that support the findings of this study are available from the corresponding author upon reasonable request.

## References

[1] M. Ogoshi , K. Inoue , K. Naruse , and Y. Takei , “Evolutionary History of the Calcitonin Gene‐Related Peptide Family in Vertebrates Revealed by Comparative Genomic Analyses,” Peptides 27 (2006): 3154–3164. 17092606 10.1016/j.peptides.2006.09.011

[2] K. Kitamura , K. Kangawa , M. Kawamoto , et al., “Adrenomedullin: A Novel Hypotensive Peptide Isolated From Human Pheochromocytoma,” Biochemical and Biophysical Research Communications 192 (1993): 553–560. 8387282 10.1006/bbrc.1993.1451

[3] E. Zudaire , A. Martinez , L. L. Ozbun , and F. Cuttitta , “Characterization of Adrenomedullin in Non‐Human Primates,” Biochemical and Biophysical Research Communications 321 (2004): 859–869. 15358106 10.1016/j.bbrc.2004.07.032

[4] K. Takahashi , K. Ohba , and K. Kaneko , “Ubiquitous Expression and Multiple Functions of Biologically Active Peptides,” Peptides 72 (2015): 184–191. 25868673 10.1016/j.peptides.2015.04.004

[5] T. Eto , J. Kato , and K. Kitamura , “Regulation of Production and Secretion of Adrenomedullin in the Cardiovascular System,” Regulatory Peptides 112 (2003): 61–69. 12667626 10.1016/s0167-0115(03)00023-5

[6] S. Sugo , N. Minamino , H. Shoji , et al., “Interleukin‐1, Tumor Necrosis Factor and Lipopolysaccharide Additively Stimulate Production of Adrenomedullin in Vascular Smooth Muscle Cells,” Biochemical and Biophysical Research Communications 207 (1995): 25–32. 7857273 10.1006/bbrc.1995.1148

[7] R. Schönauer , S. Els‐Heindl , and A. G. Beck‐Sickinger , “Adrenomedullin—New Perspectives of a Potent Peptide Hormone,” Journal of Peptide Science 23, no. 7–8 (2017): 472–485. 28150464 10.1002/psc.2953

[8] K. Kitamura , J. Sakata , K. Kangawa , M. Kojima , H. Matsuo , and T. Eto , “Cloning and Characterization of cDNA Encoding a Precursor for Human Adrenomedullin,” Biochemical and Biophysical Research Communications 194 (1993): 720–725. 7688224 10.1006/bbrc.1993.1881

[9] T. Eto , “A Review of the Biological Properties and Clinical Implications of Adrenomedullin and Proadrenomedullin N‐Terminal 20 Peptide (PAMP), Hypotensive and Vasodilating Peptides,” Peptides 22 (2001): 1693–1711. 11754955 10.1016/s0196-9781(01)00513-7

[10] N. Hirayama , K. Kitamura , T. Imamura , J. Kato , Y. Koiwaya , and T. Eto , “Secretion and Clearance of the Mature Form of Adrenomedullin in Humans,” Life Sciences 64 (1999): 2505–2509. 10403510 10.1016/s0024-3205(99)00208-8

[11] R. Pio , A. Martinez , E. J. Unsworth , et al., “Complement Factor H Is a Serum‐Binding Protein for Adrenomedullin, and the Resulting Complex Modulates the Bioactivities of Both Partners,” Journal of Biological Chemistry 276 (2001): 12292–12300. 11116141 10.1074/jbc.M007822200

[12] N. G. Morgenthaler , J. Struck , C. Alonso , and A. Bergmann , “Measurement of Midregional Proadrenomedullin in Plasma With an Immunoluminometric Assay,” Clinical Chemistry 51 (2005): 1823–1829. 16099941 10.1373/clinchem.2005.051110

[13] J. Gille , H. Ostermann , A. Dragu , and A. Sablotzki , “MR‐proADM: A New Biomarker for Early Diagnosis of Sepsis in Burned Patients,” Journal of Burn Care & Research 38 (2017): 290–298. 28221298 10.1097/BCR.0000000000000508

[14] G. Elke , F. Bloos , D. C. Wilson , P. Meybohm , and Group SCCT , “Identification of Developing Multiple Organ Failure in Sepsis Patients With Low or Moderate SOFA Scores,” Critical Care 22, no. 1 (2018): 147, 10.1186/s13054-018-2084-z. 29871660 PMC5989334

[15] D. C. Wilson , J. C. Schefold , J. Baldira , T. Spinetti , K. Saeed , and G. Elke , “Adrenomedullin in COVID‐19 Induced Endotheliitis,” Critical Care 24 (2020): 411. 32646523 10.1186/s13054-020-03151-7 PMC7347255

[16] E. Sozio , N. A. Moore , M. Fabris , et al., “Identification of COVID‐19 Patients at Risk of Hospital Admission and Mortality: A European Multicentre Retrospective Analysis of Mid‐Regional Pro‐Adrenomedullin,” Respiratory Research 23 (2022): 221. 36031619 10.1186/s12931-022-02151-1 PMC9420187

[17] G. Montrucchio , G. Sales , F. Rumbolo , et al., “Effectiveness of Mid‐Regional Pro‐Adrenomedullin (MR‐proADM) as Prognostic Marker in COVID‐19 Critically Ill Patients: An Observational Prospective Study,” PLoS One 16 (2021): e0246771. 33556140 10.1371/journal.pone.0246771 PMC7870047

[18] L. García de Guadiana‐Romualdo , M. Martínez Martínez , M. D. Rodríguez Mulero , et al., “Circulating MR‐proADM Levels, as an Indicator of Endothelial Dysfunction, for Early Risk Stratification of Mid‐Term Mortality in COVID‐19 Patients,” International Journal of Infectious Diseases 111 (2021): 211–218. 34461254 10.1016/j.ijid.2021.08.058 PMC8400460

[19] F. Leonardis , M. Minieri , M. S. Lia , et al., “Early Predictive Value of MR‐proADM in Critically Ill Patients With Covid‐19: An Observational Study in the Emergency Department,” Journal of Emergency Medicine and Care 4 (2021): 103.

[20] K. Saeed , J. M. Legramante , S. Angeletti , et al., “Mid‐Regional Pro‐Adrenomedullin as a Supplementary Tool to Clinical Parameters in Cases of Suspicion of Infection in the Emergency Department,” Expert Review of Molecular Diagnostics 21 (2021): 397–404. 33736553 10.1080/14737159.2021.1902312

[21] J. Gonzalez Del Castillo , C. Clemente‐Callejo , F. Llopis , et al., “Midregional Proadrenomedullin Safely Reduces Hospitalization in a Low Severity Cohort With Infections in the ED: A Randomized Controlled Multi‐Centre Interventional Pilot Study,” European Journal of Internal Medicine 88 (2021): 104–113. 33906810 10.1016/j.ejim.2021.03.041

[22] N. Moore , R. Williams , M. Mori , et al., “Mid‐Regional Pro‐Adrenomedullin (MR‐proADM), C‐Reactive Protein (CRP) and Other Biomarkers in the Early Identification of Disease Progression in Covid‐19 Patients in the Acute NHS Setting,” Journal of Clinical Pathology 76 (2023): 400–406. 34996755 10.1136/jclinpath-2021-207750

[23] G. Elke , F. Bloos , D. C. Wilson , et al., “The Use of Mid‐Regional Proadrenomedullin to Identify Disease Severity and Treatment Response to Sepsis – A Secondary Analysis of a Large Randomised Controlled Trial,” Critical Care 22, no. 1 (2018): 79, 10.1186/s13054-018-2001-5. 29562917 PMC5863464

[24] C. Cheyuo , W. L. Yang , and P. Wang , “The Critical Role of Adrenomedullin and Its Binding Protein, AMBP‐1, in Neuroprotection,” Biological Chemistry 393 (2012): 429–439. 22628306 10.1515/hsz-2012-0103

[25] M. Sisakht , Z. Khoshdel , A. Mahmoodazdeh , S. M. Shafiee , and M. A. Takhshid , “Adrenomedullin Increases cAMP Accumulation and BDNF Expression in Rat DRG and Spinal Motor Neurons,” Iranian Journal of Basic Medical Sciences 24 (2021): 978–985. 34712429 10.22038/ijbms.2021.54796.12289 PMC8528252

[26] B. Kis , H. Kaiya , R. Nishi , et al., “Cerebral Endothelial Cells Are a Major Source of Adrenomedullin,” Journal of Neuroendocrinology 14 (2002): 283–293. 11963825 10.1046/j.1365-2826.2002.00778.x

[27] O. Hurtado , J. Serrano , M. Sobrado , et al., “Lack of Adrenomedullin, but Not Complement Factor H, Results in Larger Infarct Size and More Extensive Brain Damage in a Focal Ischemia Model,” Neuroscience 171, no. 3 (2010): 885–892. 20854881 10.1016/j.neuroscience.2010.09.021

[28] L. Ochoa‐Callejero , A. Pozo‐Rodrigálvarez , R. Martínez‐Murillo , and A. Martinez , “Lack of Adrenomedullin in Mouse Endothelial Cells Results in Defective Angiogenesis, Enhanced Vascular Permeability, Less Metastasis, and More Brain Damage,” Scientific Reports 6 (2016): 33495. 27640364 10.1038/srep33495 PMC5027589

[29] A. P. Fernandez , J. S. Masa , M. A. Guedan , H. S. Futch , and R. Martínez‐Murillo , “Adrenomedullin Expression in Alzheimer's Brain,” Current Alzheimer Research 13, no. 4 (2016): 428–438, 10.2174/1567205013666160229112725. 26923268

[30] H. Ferrero , I. M. Larrayoz , E. Martisova , et al., “Increased Levels of Brain Adrenomedullin in the Neuropathology of Alzheimer's Disease,” Molecular Neurobiology 55 (2018): 5177–5183. 28866832 10.1007/s12035-017-0700-6

[31] M. Honda , S. Nakagawa , K. Hayashi , et al., “Adrenomedullin Improves the Blood‐Brain Barrier Function Through the Expression of Claudin‐5,” Cellular and Molecular Neurobiology 26 (2006): 109–118. 16763778 10.1007/s10571-006-9028-x PMC11520619

[32] P. Caruhel , C. Mazier , J. Kunde , N. G. Morgenthaler , and B. Darbouret , “Homogeneous Time‐Resolved Fluoroimmunoassay for the Measurement of Midregional Proadrenomedullin in Plasma on the Fully Automated System B.R.A.H.M.S KRYPTOR,” Clinical Biochemistry 42 (2009): 725–728. 19318039 10.1016/j.clinbiochem.2009.01.002

[33] CLSI , “Evaluation of Precision of Quantitative Measurement Procedures; Approved Guideline,” in CLSI Document EP05‐A3, 3rd ed. (Clinical and Laboratory Standards Institute, 2014).

[34] O. Porzio , Determinazioni Immunometriche Nei Liquidi Biologici (ELAS Ligandassay, 2021).

